# Value-based healthcare translated: a complementary view of implementation

**DOI:** 10.1186/s12913-018-3488-9

**Published:** 2018-09-03

**Authors:** Christian Colldén, Andreas Hellström

**Affiliations:** 1 Department of Technology, Management, and Economics, Chalmers University of Technology, Gothenburg, Sweden; 2 Department of Psychotic Disorders, Sahlgrenska University Hospital, Gothenburg, Sweden

**Keywords:** Implementation, Translation, Contextualization, Ambiguity, Value-based health care, Insider research, CFIR, Health care management, Management innovation

## Abstract

**Background:**

Interest in the implementation of various innovations (e.g. medical interventions and organizational approaches) has increased rapidly, and management innovations (MIs) are considered particularly complex to implement. In contrast to a traditional view that innovations are *implemented*, some scholars have promoted the view that innovations are *translated* into contexts, a view referred to as translation theory. The aim of this paper is to investigate how a translation theory perspective can inform the Consolidated Framework of Implementation Research (CFIR) to increase understanding of the complex process of putting MIs into practice. The empirical base is a two-year implementation of the MI *Value-Based Health Care* (VBHC) to a psychiatric department in a large Swedish hospital.

**Methods:**

In this longitudinal case study, a qualitative approach was applied using an insider researcher with unique access to data, who followed the implementation starting in 2015. Data sources includes field notes, documents, and audio recordings of meetings and group reflections which were abridged into an event data file structured by CFIR domains. In a joint analysis, an outsider researcher was added to strengthen the analysis and mitigate potential bias.

**Results:**

Two themes were identified, for which CFIR did not satisfactorily explain the findings. First, the *intervention characteristics* (i.e. the content of the MI) were modified along the process and, second, the *process* did not follow predefined plans. However, the project was still perceived to be successful by internal and external stakeholders.

**Conclusions:**

The paper proposes three ways in which translation theory can inform CFIR when applied to MIs: 1) strength of evidence is not as important for MIs as for medical and technical innovations; 2) adaptability of the MI can be emphasized more strongly, and 3) it can be more fruitful to view implementation as a dynamic process rather than seeing it as a matter of planning and execution. For managers, this implies encouragement to seize the opportunity to translate MIs to fit their organization, rather than to aim to be true to an original concept.

## Background

Over the past few decades, interest in the implementation of various innovations within the health care sector has grown rapidly [1]. In order to put innovations into practice and achieve improved outcomes in new settings, the process of implementation is critical [2]. However, implementation is difficult, and the challenges involved in putting innovations into practice is a frequent topic of journals and conferences for researchers and practitioners [3–5].

A number of models have been developed to increase understanding of implementation and guiding implementation projects [6–9]. The Consolidated Framework of Implementation Research (CFIR) [10] is synthesized from a number of such models, and has been used for a large variety of innovations and settings [11]. CFIR and other models have helped both researchers and practitioners to better understand the challenges of implementation of various innovations, and is one of the most recognized frameworks for implementation [12]. Management innovations (MIs) are considered even more complex to implement than technical or medical innovations [13]. However, CFIR (like most implementation models) makes no clear distinction between MIs and other innovations.

Greenhalgh et al. [14] defined innovation as *“a novel set of behaviors, routines, and ways of working that are directed at improving health outcomes, administrative efficiency, cost effectiveness, or users’ experience and that are implemented by planned and coordinated actions.”* Through an extensive literature review of diffusion of innovations in health care, they found a clear dominance of studies on short-term adoption of simple innovations, and only a few on more complex innovations. Further, their review showed that no single factor can explain why an implementation succeeds or fails – instead, it depends on dynamic interactions between factors. This is true for specific technical innovations, and even more so for innovations like MIs [15]. Several scholars have studied MIs, under labels such as *organizational innovations* [13], *administrative innovations* [16], *management concepts* [17], and *management ideas* [18]. In this paper, the term *management innovation* (MI) is used, defined as a *“management practice, process, structure, or technique that is … intended to further organizational goals”* [19]. MIs are typically complex and context-dependent; therefore, boundaries between MIs and their surroundings may be indistinct [20, 21].

MIs differ from medical and technical innovations in that they often contain a high degree of conceptual ambiguity that makes it difficult to pinpoint their exact meaning [13]. Hence, they can be interpreted in different ways by different individuals. In addition, this ambiguity allows for adaptation to different contexts [22], and it has been argued that research on MIs should focus on *how* organizations define MIs and put them into practice [16]. Adopting a similar view, scholars studying fashions and spread of MIs have argued that rather than being *implemented*, MIs can be seen as *translated* [18, 23, 24] into a setting. This view – which is referred to in this paper as *translation theory* – highlights the influence of humans and human actions when an MI goes from idea to reality.

The purpose of this paper is to investigate how a translation theory perspective can inform CFIR in order to increase understanding of the complex process of putting MIs into practice. We present a case of implementation of the contemporary MI *value-based health care* (VBHC). VBHC can be seen as a typical example of an MI with its novel set of management practices intended to improve health outcomes and efficiency, and many organizations currently struggle to implement this MI [25–30].

## Theoretical background

As indicated above, *implementation* and *translation* refer to the same process but imply different views on the phenomenon. Below, the CFIR, representing an implementation view, is presented, followed by a section on translation theory in relation to MIs. Finally, VBHC is described, as the MI in focus in the current study.

### Consolidated framework for implementation research

CFIR was first presented in 2009 [10] as a conceptual framework intended to guide implementation research pre-, during, and post-implementation, for both technical and administrative interventions (in this paper, MIs). A total of 39 constructs affecting the implementation process are proposed, which are listed in full in the original work [10] and are grouped into five domains:

1. Intervention characteristics
2. Outer setting
3. Inner setting
4. Characteristics of individuals
5. Process

CFIR *“is applicable to a wide range of interventions, settings, and research designs”* [11] and has been used for studies related to a range of interventions; for example, programs for weight management and physical activity [31], tumour screenings [32], supportive housing [33], blood pressure management [34], and more general implementations of evidence-based practices in public health agencies [35]. Notably, a common denominator is that interventions are limited to specific medical or technical methods or procedures. Applications of CFIR to more general MIs are rare, but some have been presented; for example, for a reimbursement and benchmarking system [36], a health care delivery redesign project [37], a “patient-aligned toolkit” [38], and primary care leadership [39]. Notably, though, Damschroder et al. ([10] (Additional file 4)) argued that even though CFIR is intended for all kinds of innovations, *“administrative interventions tend to be more complex and difficult to implement”*.

### Translation of management innovations

An influential perspective in studying the spread of innovations is the *diffusion of innovation theory* [14, 40–43]. Within this, the “why” and “how” of the way in which organizations adopt different innovations have been issues of interest [44, 45]. This rational view has suggested that organizations adopt MIs because they need to realign the organization with a changing environment [46]. That is, adoption might be driven by a need to perform better and be more competitive. However, adoption of MIs may also be a response to institutional pressures – such as laws, norms, standards, policies, and current management fashion – in order to create an image of being modern and to retain legitimacy, rather than for reasons connected to pure effectiveness [44, 47]. Hence, the reasons for adopting MIs are not always rational.

Further, Rogers [40] argued that *“previous diffusion research … generally stopped short of investigating implementation. Once a decision to adopt in an organization has been made in an organization, implementation does not always follow…”* Later research has looked more deeply into aspects of implementation processes [48–50], and some scholars have rejected the term “implementation” [51] in favour of “translation” [18, 23, 52]. For example, Latour [24] argued that the term “translation” better recognizes the fact that the spread of innovations in time and space “*is in the hands of people.*” Røvik [18, 52] further described the different sub-processes included in the translation of MIs to certain contexts, but also stressed that these sub-processes are in practice often overlapping. Therefore, Røvik [18] called for more research on how translation processes are related in various contexts, noting that “*translation may be a key to understanding how organizational resistance toward certain ideas either can be blocked … or carefully handled*” [18].

However, conditions for the translation process are affected by characteristics of the MI. One such characteristic is the MI’s *interpretative viability* [22]; MIs often have no material component and are characterized by a certain degree of conceptual ambiguity, which creates room for interpretation [13]. This room for interpretation increases the chance that the MI will gain popularity, since the MI can be described and understood differently by different managers or organizations, who then more easily apply it to their own situations. Such different interpretations may obscure conflicting goals [53]. Thus, promises of improvements (which are normally attached to MIs) make the MI attractive, while its vagueness means that potential users can eclectically select those elements that appeal to them [22].

### Value-based health care

One frequently considered MIs in recent years is VBHC [54]. The core of this MI is the definition of value as outcomes that matter to the patient in relation to the costs of delivering care. Central claims include the fact that health care systems of today are fragmented, inefficient, and lacking transparency for patients to make informed choices about their care [55, 56]. In short, VBHC advocates a change to a more coherent health care system, comprising six interdependent elements:

1. organize care into integrated practice units;
2. measure outcomes and costs for every patient;
3. reimburse through bundled payments for full care cycles (from onset to end-stage);
4. integrate care across different facilities;
5. expand services with the best outcomes across geography; and
6. create enabling information technology platforms.

Thus, VBHC considers the overarching perspectives of health care systems, rather than the actual care operations [57], aiming to improve both health outcomes and cost effectiveness by new ways of working. Hence, it is a typical example of an MI. Also, VBHC is gaining substantial interest in relation to several patient groups (including cancer [58], spinal disorders [59], and people living with complex long-term conditions [60]) and in many parts of the world (such as the US [61], Europe [5, 62], and Asia [63]). However, the label VBHC is sometimes used without deeper understanding of the original concept [64], though some promising results have also been presented. For example, the focus in VBHC on patient-reported health outcomes has led to reduced nausea after surgery, and the focus on integration of care within and across facilities has led to an increase of available beds for patients in need of admission [65].

## Method

In this paper, we present a longitudinal case study of a two-year VBHC implementation project in a psychiatric department in Sweden. To capture in depth the complex interactions within the process of implementation (or translation, depending on which view is adopted), the study is based on rich qualitative data from an insider researcher with unique access to and understanding of the context. As Greenhalgh [45] argued, *“These interactions are unlikely to be elucidated by the randomized controlled trial design that still dominates much health technology research. Rather, we need studies that are interdisciplinary, nondeterministic, locally situated, and designed to examine the recursive relationship between human action and the wider organizational and system context.”*

### Setting

The study setting is the Department of Psychotic Disorders at the Sahlgrenska University Hospital in Gothenburg, Sweden. The hospital has approximately 16,000 employees and 2000 beds. The Department of Psychotic Disorders is one out of approximately 50 departments and has 400 employees providing both in- and out-patient care for 2600 patients with schizophrenia and schizophrenia-like disorders. The setting was selected based on the department’s engagement in improvement work and our unique access to data, as one of the authors has held a position as section manager within the department since 2011. An insider role gives a deep understanding of context and culture in the organization. By conducting research in a group that he is also a member of, the researcher also shares identity, language, and experiences with the participants [66]. This membership role gives the researcher a certain amount of legitimacy [67], which may allow for greater depth to the data gathered. However, this closeness to data also inherently brings a risk of bias. To avoid such bias and take advantage of both an insider and an outsider perspective, data was critically reflected on via a joint analysis between the two authors, where the second author did not have an a priori understanding of the setting. This is in line with Breen’s [68] argument that collaboration between an insider and an outsider can *“balance the advantages of both positions while minimising the disadvantages of each.”*

### Data collection

An insider researcher (the first author of this paper) followed the MI implementation for two years to obtain a deep understanding of the process [69]. The researcher was one of two cooperating project leaders, and as section manager in the department he had access to data about both actions and contextual factors. Documentation and field notes were collected from meetings with the project group, steering group, and reference group, as well as from other related meetings and events (such as dialogues with a foreign professor and an experienced clinical leader, and also joint meetings for all project groups currently implementing VBHC at the hospital) throughout the implementation project. The main study objects are further described in Table 1. Notes, including immediate reflections, were made in close temporal relation to events to allow for later distinctions between direct and condensed interpretations [70]. Relevant documents (including guidelines and mailings from meetings) were also collected continuously between meetings. Furthermore, the first author regularly invited the project group to jointly reflect on ongoing processes immediately after ordinary meetings so as to generate and capture knowledge in cooperation with organization members, since *“capturing multiple and diverse interpretations adds to a deeper, richer picture of the issue at hand”* [69]. These 5–15 min sessions were also audio-recorded in order to allow for deeper analysis at a later stage [71]. Table 2 provides an overview of the data.

**Table 1:** Study objects within the research project and their functions in practice and research

Study object	Group members	Role in practice	Rationale for study
Project group	Two cooperating project leaders, one care developer, and administrative support	Led the local implementation project, developed material as decision basis, planned and coordinated pilot projects, and led meetings with the steering and reference groups and other stakeholders.	The core of the implementation process, where most of the actual work was done, and information and actual power was concentrated.
Steering group	Seven members including the head of department, four first-line managers, one operations coordinator at department level, and one quality controller at division level.	Made all strategic decisions, based on the material produced by the projects group and discussions together with the reference group.	Constituted a managerial perspective from within the context and had important power over strategic decisions.
Reference group	Twelve employees with different professions and from different units within the department, chosen to include as many perspectives as possible.	Gave feedback on decision basis material and discussed questions raised by the project group to help the project and steering groups to make strategic decisions.	Provided important information about the inner context and affected both the content and the process of implementation.
Internal consultants	This hospital-level unit consisted of 6–10 consultants specialized in e.g. logistics, implementation, and quality assurance, trained in VBHC. Two consultants were involved in the project group for schizophrenia.	Controlled the implementation of VBHC initiative at hospital level, provided implementation support to project groups in different departments, and arranged joint meetings for all active project groups.	Important stakeholder, controlling the framework for implementation, hence constituting an important part of the outer context and also provided an outsider perspective assessing the level of success of the local implementation project.

**Table 2:** Overview of collected data

Forum	Type of documentation	n
Project group meetings	Field notes	35
	Audio recordings (full)	4
Joint meetings with steering and reference groups	Field notes	9
	Audio recordings (full)	2
Other related meetings and events	Field notes	18
Guidelines, documentation, mailings, etc. from and in between meetings and events	Documents	53
Reflective discussions with project group	Audio recordings	13

All involved consultants and group members (i.e. all members of the project, steering, and reference groups) were informed in advance about the research study and given the opportunity to decline participation or being recorded, or choose to be anonymously quoted.

### Data analysis

Data from the various sources were continuously gathered in an event data file (as inspired by Maxwell [72]) consisting of shorter memos for each event and monthly summaries structured according to the CFIR domains (intervention characteristics, inner setting, outer setting, characteristics of the individuals, and process [10]). Thereafter, an outsider researcher with previous experiences in research on implementation of MIs in health care settings but no preunderstanding of the studied organization (the second author of this paper) was brought in for analysis of the case data. Starting from the event data file, the analysis was conducted first independently and then jointly by the two authors to diminish the risk of potential bias, strengthen the analysis, and allow for a constructive dialogue [68, 73]. In the joint analysis, the events and content of issues under debate were reviewed and analysed using CFIR and translation theory, respectively. For two themes, the analyses differed depending on what framework was applied, and for these themes translation theory was seen to provide a complementary view to CFIR. One theme concerned *what* was being implemented and the other *how* it was implemented. Referring to the CFIR domains, the themes were named *Intervention characteristics* and *Process*. These CFIR domains include a total of 16 constructs, as described in Table 3.

**Table 3:** CFIR constructs for the domains of Intervention characteristics and Process. Adapted from Damschroder et al. [10]

Domain	Construct	Description
Intervention Characteristics	Intervention source	Key stakeholders’ perceptions of whether the intervention developed within or outside of the organization.
	Evidence strength & quality	The perception of stakeholders regarding the validity of evidence in support of the intervention’s potential to bring about the desired outcomes.
	Relative advantage	Advantage of the intervention over alternative solutions in the eyes of stakeholders.
	Adaptability	The degree to which the intervention can be transformed or customized to fit with local needs.
	Trialability	The potential for testing the intervention in small, reversible steps.
	Complexity	Perceived intricacy of the implementation due to scope, disruptiveness, profoundness, number of stakeholder groups, etc.
	Design quality and packaging	Perception regarding how well compiled and presented the intervention is.
	Cost	Costs associated with the implementation and use of the intervention.
Process	Planning	The quality of a pre-defined method or scheme for the implementation, and the degree to which it is applied.
	Engaging	Attracting and involving key individuals (listed below) in strategies including social marketing, training, role modelling, etc.
	- Opinion leaders	Organization members with formal or informal influence on colleagues.
	- Implementation leaders	Individuals in the organization who have been formally appointed as responsible for the implementation.
	- Champions	Dedicated individuals who are passionate about the intervention.
	- External change agents	Individuals who are not part of the organization but formally affect or facilitate implementation positively.
	Executing	Accomplishing the implementation according to the plans made in advance.
	Reflecting & evaluating	Feedback about the implementation progress, for example through regular personal and team reflections on progress and experiences.

## Results

This section presents an overview of the case study, followed by a description of two cross-cutting themes based on CFIR domains of particular relevance.

### The introduction of VBHC

The hospital’s introduction of VBHC was initiated in 2013 in cooperation with an external, international consulting firm, as per the process described by Nilsson et al. [5]. Initially, the implementation focused mainly on two elements of the original VBHC concept: *measure outcomes and costs for every patient* (which was divided into two parts with separate focuses: *measurements* and *adoption of a patient’s view as point of departure*), and *benchmarking* (part of the element *expand services with the best outcomes across geography*) [25]. In addition, the element *integrate care across different facilities* was considered by implementing VBHC for diagnostic groups bridging different departments organized by medical specialty. However, facilities outside of the hospital (such as municipal units and primary care) were not integrated. The implementation initiative was rolled out in waves of four to six diagnostic groups at a time, and the external consultant firm was replaced by an internal consulting organization.

The schizophrenia patient group officially started its implementation of VBHC in early 2016, preceded by a six-month preparation phase. A project group and two cooperating project leaders, of whom one was also a researcher (the first author of this paper), was appointed by the head of department. Two sets of organizational members were strategically chosen to form a steering group and a reference group, respectively, and a plan for the project was developed, all in line with guidelines from the internal VBHC consulting organization.

From the preparation phase on, some of the central activities included searching for relevant outcome measures from the perspective of the patient, mapping current processes and routines for measurements, and searching for other centres suitable for benchmarking and inspiration. Several pilot projects were also started in order to test measures and new routines for data collection. In April 2016 the implementation phase ended, and a set of measures (most of which were process measures) was presented, forming an initial scorecard, together with a future scorecard including measures that were considered better in terms of reflecting actual outcomes, but were not feasible at the time due to underdeveloped IT systems and routines for data collection. A plan for further development and improvements, and integration of the new scorecard into the existing management system, was also presented. In concluding meetings, the project was evaluated subjectively by group members and internal consultants at hospital level, who generally perceived the implementation project as successful since, for example, employees showed engagement in change, useful scorecards had been agreed upon, and promising pilot projects had been initiated.

### Theme one: Intervention characteristics

The first identified theme in relation to CFIR domains concerns perceptions of the content of VBHC, and the strategic choices made by project leaders in relation to it. When the project group met with the reference group as well as professionals from different units within the organization, it became evident that many professionals were somewhat critical towards measurements due to previous experiences of time-consuming coding and reporting with no (for them) useful feedback. At the time there was also relatively widespread criticism in media of productivity measures connected to “new public management.” Simultaneously, VBHC was perceived positively, even though the professionals’ understanding of it was very superficial and the interpretation of “value” differed, as described in an earlier study in the same setting [57]. For example, in discussing what outcome measures to use, some individuals advocated aspects such as “severity of symptoms” and “survival” (that is, life expectancy), whereas others emphasized “participation in society,” thereby demonstrating different perspectives. Still others indicated that value is such a fuzzy concept that there is no point in refining the measurement, and instead promoted a simple visual-analogue scale for current mood in general. All these views fitted the description of VBHC and allowed individuals to attach their own views to it, up to the point where definite choices were to be made. In sum, individuals with different points of view could all attach hope to the new fuzzy concept VBHC.

Furthermore, VBHC was originally presented as a general approach that is applicable to all medical conditions and specialties and aims to move focus away from technical and economic matters (of interest mostly to administrators) to, instead, aspects of care that matter to patients (and to professionals with direct patient contact). However, the concrete implications for specific settings were also fuzzy. In this case, although the audience to a large extent comprised medical specialists, scientific evidence played no prominent role in the argument for adoption. Rather, the hospital CEO and other advocates referred to examples of other successful health care organizations and common sense (for example, “comparisons and exchange of experiences make us learn from each other” and “working together with patients improves the quality of care”).

Measurements are a vital part of VBHC. At the same time, the project group had an understanding of the context that some professionals were sceptical and tired of measurements. Therefore, in order to forestall organizational resistance and gain acceptance among professionals, the project group first focused on reducing measurements that professionals perceived as meaningless and time consuming. For example, a shortlist was developed, consisting of 14 activity codes that should be registered when performed, replacing an earlier list of approximately 120 codes which had been used inconsistently. In addition, in order to further improve the data handling – and hence convenience for the health care professionals – the project group started pilot projects for a new database solution that would simplify data input, output (to quality registers), and feedback (to patients, staff, and managers). The strategy was intended to prove the usefulness of measures and use the assumed enthusiasm both from within the organization and from higher management as leverage against the IT organization, which was sceptical to separate local IT solutions and therefore acted as an obstacle. In this way, the project leaders actively amplified the *relative advantage* (italics indicate CFIR constructs, see Table 3) of the MI and, to some extent, shifted the impression of the *source of intervention* internally – that is, making the changes appear to organizational members as locally invented. Furthermore, by decomposing VBHC into smaller pilot projects, which were more or less tightly coupled with the original concept but all presented under the VBHC umbrella, the *trialability* of the MI was enhanced, since each pilot project could be modified and/or withdrawn separately. This “lack of faithfulness” to the original VBHC concept (in terms of choosing only some elements and connecting other changes to VBHC) can be seen as an effort to exploit of the *adaptability* of the MI to the greatest extent possible.

### Theme two: Process

**Fig. 1:**
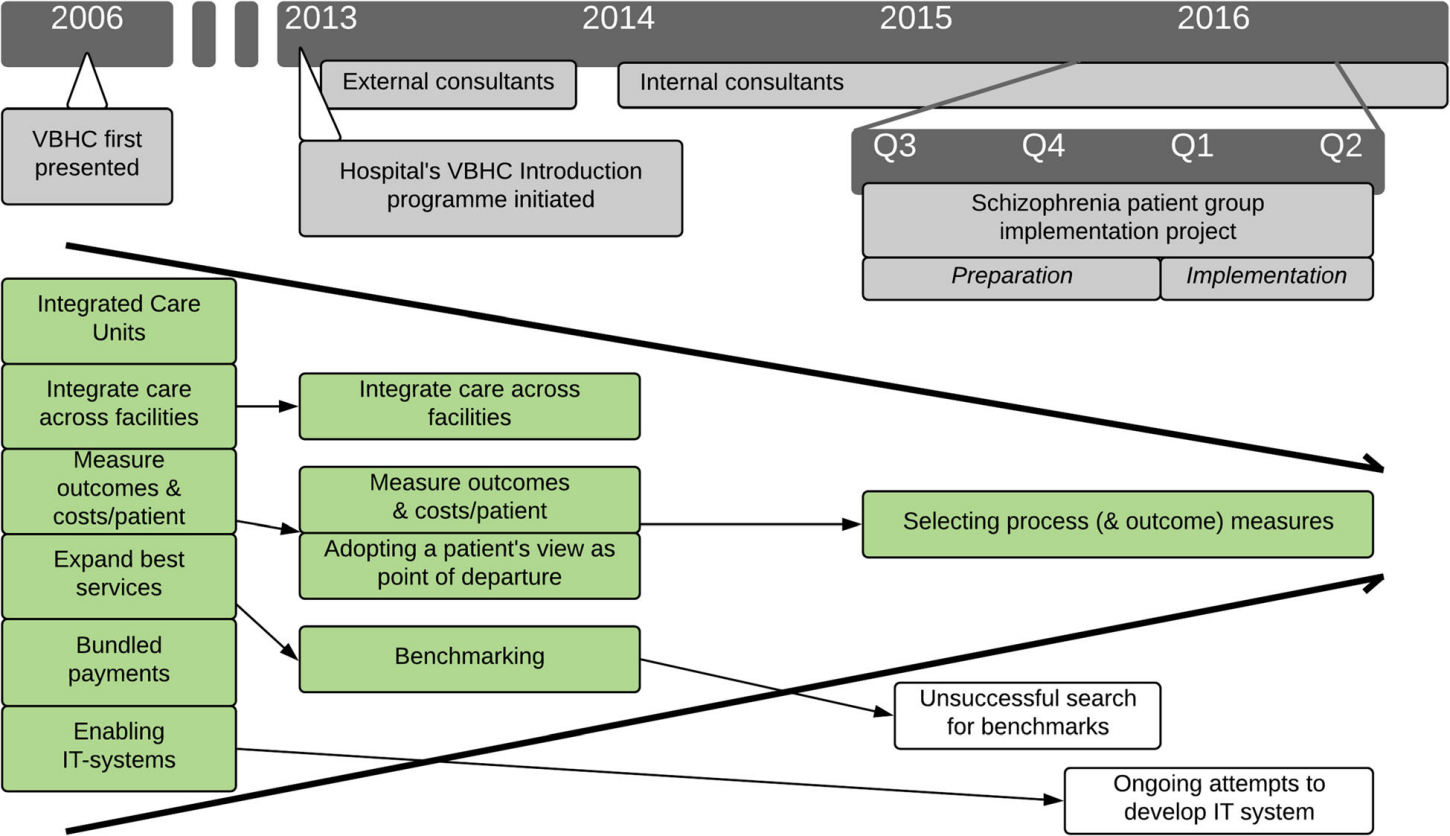
Timeline of VBHC introduction to the hospital, and local implementation to the schizophrenia patient group. Note: Figure includes elements of VBHC (green) remaining at different points in time. White boxes represent elements included in the local implementation but not accomplished. Thick lines point to the narrowing of the original concept’s scope

Starting from the originally presented concept of VBHC, the scope of the implementation effort was narrowed in several steps, as illustrated in Fig. 1. First, the external consulting firm hired to facilitate the initial implementation focused on measurement of outcomes (preferably patient-reported), benchmarking (that is, competition based on outcomes), and involvement of patients, but left out payment models and organization in integrated practice units, among other elements. Next, the internal consultants further lowered the ambition of staying true to the original concept by accepting more process measures, as long as these were putatively connected to outcomes. Development of supportive IT systems was also postponed due to technical and organizational obstacles that the project leader driving technical solutions considered too complex to overcome in the short term. Thus, even though structured guidelines (that is, *planning*) for the local implementation projects were developed, no overarching plan for implementation of the entire VBHC concept was presented, and the *execution* of implementation did not follow the planning as suggested by CFIR.

The local implementation project followed a similar development path, with more and more elements of VBHC being left out. Initial ambitions included a radical change to focus on (patient-reported) outcomes, extensive patient involvement, supportive IT systems, and international competition based on outcomes, inspired not only by the hospital implementation initiative but also by the original sources [54]. However, as the ideas were introduced to practice, most parts were left out. No international centres using comparable continuous outcomes monitoring were found; IT development, while not abandoned, was heavily delayed by external obstacles; and even though patient involvement occurred, the goal in relation to this was scaled down. The only tangible result of the implementation project that remained was a set of partly new measures and improved routines for collection and reporting of data – a significant operational improvement, but far from the original concept as a whole. Here, a *plan* was outlined from a higher authority, but the *execution* failed as obstacles arose. New plans were then made iteratively by the project leaders, which partly addressed the problems and partly included associated developments driven by operational needs. These partial and evolving plans were executed with greater faithfulness to the plans. Aspects of *engaging* were also included in these local plans, and were at least partially successful, primarily for individuals who were directly involved in project-associated groups and pilot projects. *Reflecting and evaluating* was also planned for and conducted iteratively, but the focus was on acceptance and operational improvement, rather than on implementation of the VBHC concept. Hence, structured *planning* and subsequent *execution* of implementation is not a description that fits the case data well.

## Discussion

### Analysis from a translation theory perspective

As described above, two themes emerged in the data – intervention characteristics and process – for which translation theory was seen to provide a complementary view to CFIR. The analysis of these two themes according to translation theory is presented below.

#### Theme one: Intervention characteristics

Adopting the lens of translation theory, we see that management fashions (that is, initiatives that are discussed in positive terms in the outer setting and used by successful organizations [74, 75]) is a more important factor in convincing the organization to adopt the MI compared to the strength of evidence, which is more emphasized by CFIR [10]. Moreover, VBHC is described in general terms at a high level of abstraction and is centred on the ambiguous concept of value; hence, in terms of CFIR constructs, it is highly adaptable. According to the nomenclature of translation theory, VBHC has large interpretative viability [22], which allowed the project group to tailor the MI to the (individuals within the) context. Hence, beliefs and promises were boosted and used to create a vision of a high relative advantage [10] of VBHC versus the current system. Moreover, the interpretative viability of the MI was used to fill the change initiative with content that was important to the organization, rather than true to the original VBHC concept.

#### Theme two: Process

Interpretative viability [22] played a central role within this theme. The content of the VBHC implementation project was pragmatically selected and adapted in several steps. In other words, VBHC was iteratively translated [23]. First, at the hospital level, only the parts of VBHC that concerned the internal affairs of the hospital were in focus. Next, external consultants made pragmatic choices regarding aspects to emphasize, as did the internal consultants and the project group, resulting in a “funnel effect” (that is, the scope of change was increasingly tapered). Design of the health care system organization and reimbursements were questions for external authorities. The IT environment turned out to be too complex to influence in the short term. Hence, ultimately the scope of the VBHC introduction was narrowed to improved measurements and a vision of using the measures to change the focus of care development and enable comparisons with other centres to learn and compete. Nevertheless, this narrow scope appeared to the project leaders in the case as elements that could be useful for improving operations. Hence, the emphasis in the local project on selecting measures for a scorecard and on developing data-collection routines to be more credible was strengthened. Consequently, eventually only a fragment of the original VBHC concept (that is, measurements) was implemented. However, the operational outcome was promising. Even though this study did not contain any outcome measurement, the project was perceived as a success by both organization members and internal consultants (when subjectively comparing it to implementations pertaining to other patient groups), and was still labelled a VBHC implementation.

It is clear that translations [23, 24] of VBHC had a large impact on the end result. The translations were made by individuals, or groups of individuals, at different stages and organizational levels. These individuals all had different (limited) levels of influence and made choices based both on what was possible and on individual beliefs and visions. Some decisions seem to have been conscious, and others unconscious. Thus, the process of translation was heavily dependent on the individuals’ choices and actions.

### Translation theory and CFIR

The discourse on popular MIs is an important part of every organization’s environment, and affects how organizations are managed by shaping managers’ understanding of what organizations can, may, or must do [46]. Consequently, it is important to better understand how organizations act to put their MIs into practice, and what processes they go through in their progress from general idea to local routine.

Our case of implementation of VBHC illustrates that, at least for complex and ambiguous innovations such as MIs, implementation frameworks like CFIR [10] can benefit from complementary theoretical fields. In this paper, we have elaborated on the addition of translation theory, which stresses the impact of human interpretations and actions in a wider organizational context. We see three ways in which translation theory can inform CFIR.

First, the rationale for implementation of a specific MI is not always dependent on scientific evidence. In CFIR, *strength of evidence* is regarded as an influential characteristic [10], but was not prominent in the presented case. Instead, the implementation was promoted by general arguments, such as the idea that increasing focus on what is valuable for the patients enables learning from other centres and examples of successful organizations applying principles of VBHC. Nevertheless, these arguments were accepted even by scientifically trained medical specialists.

Second, the CFIR construct *adaptability* aligns with *interpretative viability* [22], but can be further emphasized and developed for implementation of MIs. The very nature of an MI, with its complex and ambiguous content, offers an interpretive viability that allows for different courses of action in different organizational entities, while still maintaining a unifying common label. In the case presented here, many core elements of the original VBHC concept [54, 56] were left out along the path of implementation. This can be seen as what Giroux [53] called pragmatic ambiguity – *“the condition of admitting more than one course of action.”* The case also illustrates that choices made by individuals, which are heavily dependent on contextual factors, have a large impact on the end result (that is, which elements of the original MI are kept). For this context-dependent dynamic process, Røvik [52] proposed the term *contextualization*, describing a *“hierarchical chain of translation.”* Røvik stated that in every step of translation in the contextualization process, elements of the MI are subjects of transforming mechanisms and can be copied, subtracted, or altered, or even new elements can be added. In the presented case, the original concept of VBHC [54, 56] proposed a change to a coherent health care system with some specific characteristics, but the version of VBHC that was actually put in place in the specific setting included only an improved system for performance measurement. Nevertheless, it was still considered a successful project by the internal consultants at the hospital and by the head of department, once again reflecting the importance of pragmatic ambiguity in a contextualization process. Hence, MIs can take on very different shapes and meanings in different organizations, or even in different parts of the same organization. Thus, it is important to consider contextualization in the implementation initiatives, to stress the importance of *adaptability*.

Third, and as a further extension of the aspect of contextualization, an instrumental view of a pre-planned implementation process is not always fruitful. CFIR [10] promotes *planning* in advance and *execution* in consistence with the plans. However, viewing an MI as being contextualized in iterative translations implies that *planning* cannot always be developed in advance and *executing* cannot always follow the plans. In our case, plans were made by both external agents and the project leaders but were not always executed accordingly. Instead, the plans were repeatedly changed and adapted, more in line with a translation process, as promoted by translation theory [22]. Thus, allowing a more emergent process that adapts to the changing context [23] may help MIs become more useful in practice by optimizing the conditions for improved operational outcomes.

This study shows that translation theory may make notable contributions to implementation science. In line with Greenhalgh al.’s [45] call for research on the implementation of complex innovations or in complex settings, a translation theory perspective is nondeterministic, locally situated, and focused on the relationship between human action and the organizational context. Inclusion of this perspective in CFIR and other implementation frameworks may improve understanding of why some implementations succeed and others do not. However, to further develop implementation frameworks such as CFIR for complex and ambiguous innovations like MIs, more research is needed on the mechanisms involved in the contextualization process, as described by Røvik [52]. In addition, this study is small and limited to a single implementation initiative. The proposed benefits of translation theory for CFIR also need to be studied further, preferably via comparative multiple case studies.

## Conclusion

In this paper, we presented a case of implementation of VBHC and analysed this through the lenses of CFIR and translation theory, respectively. The case illustrated that the original concept of VBHC, due to its interpretative viability and pragmatic ambiguity [53], was repeatedly translated and thus heavily modified during the process, in a way that can be described as contextualization [52]. We thus showed that research on implementation, and frameworks such as CFIR, can benefit from including the complementary view of translation theory when it comes to more complex and ambiguous concepts like MIs. First, strength of evidence is not as important for MIs as it is for medico-technical innovations. Second, adaptability can be emphasized more, and developed to include the concept of contextualization. Third, the view that implementation processes should best be executed in line with predefined plans or schemes is not fruitful for MIs, for which the inherent interpretative viability makes the choices of individuals crucial for what the end result of the implementation process will look like. Thus, a translation theory perspective accepts that the local application of an MI may differ from its original form and, rather, encourages managers to seize the opportunity to contextualize the MI to fit their own organizations. As Greenhalgh [45] suggested, studies that investigate the relationship between actions of humans and wider organizational systems are important to further understand implementation. More such studies are needed in the future, both in other contexts and for other MIs.

## Abbreviations

CFIR: Consolidated Framework of Implementation Research
MI: Management Innovation
VBHC: Value-Based Health Care
